# Spontaneous Clearance of Viral Infections by Mesoscopic Fluctuations

**DOI:** 10.1371/journal.pone.0038549

**Published:** 2012-06-05

**Authors:** Srabanti Chaudhury, Alan S. Perelson, Nikolai A. Sinitstyn

**Affiliations:** 1 Theoretical Division, Los Alamos National Laboratory, Los Alamos, New Mexico, United States of America; 2 Theoretical Biology & Biophysics, Los Alamos National Laboratory, Los Alamos, New Mexico, United States of America; 3 New Mexico Consortium, Los Alamos, New Mexico, United States of America; Northeastern University, United States of America

## Abstract

Spontaneous disease extinction can occur due to a rare stochastic fluctuation. We explore this process, both numerically and theoretically, in two minimal models of stochastic *viral infection* dynamics. We propose a method that reduces the complexity in models of viral infections so that the remaining dynamics can be studied by previously developed techniques for analyzing epidemiological models. Using this technique, we obtain an expression for the infection clearance time as a function of kinetic parameters. We apply our theoretical results to study stochastic infection clearance for specific stages of HIV and HCV dynamics. Our results show that the typical time for stochastic clearance of a viral infection increases exponentially with the size of the population, but infection still can be cleared spontaneously within a reasonable time interval in a certain population of cells. We also show that the clearance time is exponentially sensitive to the viral decay rate and viral infectivity but only linearly dependent on the lifetime of an infected cell. This suggests that if standard drug therapy fails to clear an infection then intensifying therapy by adding a drug that reduces the rate of cell infection rather than immune modulators that hasten infected cell death may be more useful in ultimately clearing remaining pockets of infection.

## Introduction

Deterministic models of viral infection have been successful in fitting experimental data and extracting useful parameter values [1], [2], [3], [4]. Such models are based on a large population of infected cells and virions, so they fail to capture some important stages of viral infection dynamics for which intrinsic stochastic effects play a dominant role. One of the remarkable phenomena observed in stochastic population dynamics is spontaneous extinction of a disease via a rare fluctuation. Small population sizes or heterogeneity in populations are some of the determining factors for extinction to occur. A major characteristic of disease extinction is the extinction time - the mean time in which the number of infected cells reaches zero. An estimate of the time required for the infection to be cleared can be tested during drug treatment on a patient.

The theory of disease extinction should identify parameters that are most important for determining the extinction time, and hence suggest processes that one might want to target by drug therapy to decrease the extinction time. For example, recent experimental work [5] has proposed a method that predicts the location, timing and magnitude of the immune response needed for a vaccine to eliminate persistent infection in the early stages of viral infection.

Models that describe spontaneous virus extinction can be relevant to various stages of a viral infection. Up to one third of patients with acute hepatitis C virus (HCV) infection spontaneously clear the infection [6]. Because HCV levels reach a plateau or steady-state level within the first week to two of infection [7], it is likely that patients who spontaneously clear do so from a steady state. Spontaneous clearance of HCV infection can take place in the presence of HCV specific immune responses [8], [9]. There has also been a report on chimpanzees demonstrating the clearance of HCV infection spontaneously in the absence of a detectable immune response [10]. More recent studies demonstrate the clearance of low levels of HCV in humans in the absence of HCV specific cellular immune responses [11]. These results suggest that a small population of infected cells in the human liver can be cleared spontaneously even in the absence of drug therapy or an immune response. Under drug treatment, a drug may not be delivered equally well to all infected regions in the body, as reported for HIV-1 infection [12], [13]. Thus, there may be regions with a small number of infected cells in which drug levels are low so that stochastic extinction of virus and infected cells may be the major recovery mechanism in those regions. Spontaneous disease extinction can also occur during HIV infection propagation at early stages of the disease. Before entering the blood stream, a sexually transmitted virus, such as HIV, has to diffuse through the tissue in the genital tract where it experiences a scarce supply of susceptible cells and spatial confinement in a strongly heterogeneous medium [14]. In such a situation, the infection may enter a quasi-steady state locally. This state is characterized by a balance between the rate of virus decay and replication. Spontaneous infection clearance may take place from such a quasi-steady state. In the context of HIV or HCV infection, we will assume that the total number of infected cells in the considered region, *n_I_*, is relatively small and fluctuates with time so that the size of a typical fluctuation is of the order of ∼ *n_I_^1/2^*. In the context of HCV infection, we will use kinetic parameters estimated in Rong et al. [27], while for HIV we use parameters discussed by Pearson et al. [15]. Since an infection can go extinct only due to a rare event, spontaneous infection clearance is described by the tails of the infected cell number probability distribution. With these assumptions we estimate that the critical population size for spontaneous clearance of HIV infection is of the order of *n_I_ ∼10* cells. Our estimates of the critical population size in infection with kinetic parameters of HCV demonstrate that when the number of infected cells in a region is about 40 or less, then spontaneous clearance would take place during a year.

A theoretical approach to compute fluctuation induced disease extinction time for non-viral infections was developed recently based on the so-called semi-classical path integral technique [16], [17], [18]. This method has been successfully applied to several stochastic epidemiological models such as the endemic susceptible – infected - recovered (SIR), and SI models [19], and the endemic SIS model [18] to determine the mean extinction time of a disease.

Despite the success in epidemic models, the semi-classical method has not been applied to viral infections. This can be explained partly by the relative complexity of stochastic models that involve both virus particles and infected/susceptible cells. One of our goals in this manuscript is to show that the complexity of viral infection dynamics can be effectively reduced in the semi-classical approach using a coarse-graining technique developed recently [20], [21]. We will show that coarse-graining typically reduces the models of viral infections to some of the previously studied types of stochastic SI models.

Before we discuss a general model of interactions between viruses and infected/susceptible cells, we explore in some detail two minimal stochastic models of early infection that were partly explored recently by Pearson et al. [15]. They studied the extinction probability with the assumption that the infection starts from a given number of virions and infected cells that are introduced into a body in which an extremely large number of cells are susceptible to infection; the question of spontaneous infection clearance from an endemic state was not considered. Stochastic simulations of HIV and SIV infection, performed earlier [22], [23], [24], show that during early infection, the number of virions and infected cells either both reach zero implying extinction or they both eventually reach constant quasi-steady state values at which the infection is maintained. Our semi-classical technique complements the study by Pearson et al. [15] by allowing us to consider more complex behavior such as infection extinction from a metastable state.

## Methods

### Models of Viral Infection

We will concentrate on the two models studied by Pearson et al. [15], the continuous virus production model (Model 1) and the burst model of viral production (Model 2); however, our approach can be easily extended to arbitrary systems. As in Pearson et al. [15], we assume all virions are equivalent and equally infectious and thus ignore non-infectious or defective virus particles. As discussed by Pearson et al. [15], it is not known whether viral production in HIV occurs in a burst or is continuous. Burst production may be a reasonable approximation for viral production from activated T cells, while continuous production may be more relevant for production from resting T cells or macrophages. Since HCV infection generally does not kill the cells it infects [25], production of HCV is most likely continuous.

#### Model 1 (Continuous viral production)

**Figure 1:**
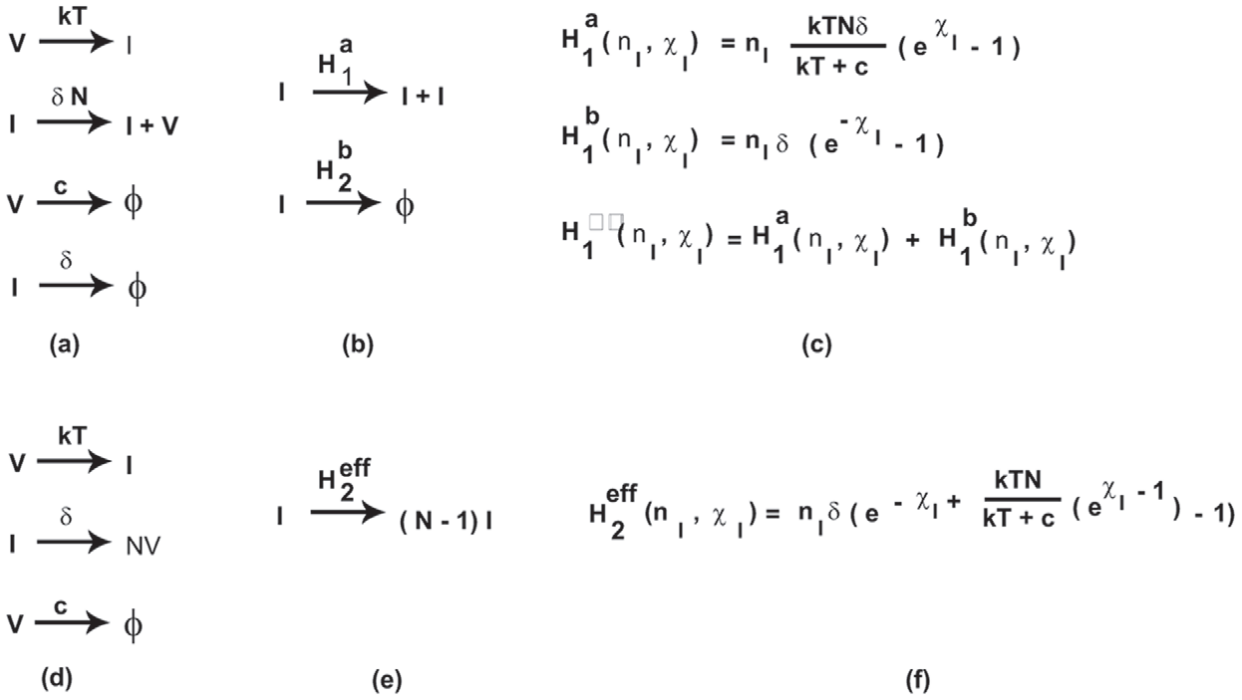
Schematic representation of viral infection models, (a) Model 1 (b) Comparison with simple SIS model (c) Comparison of effective Hamiltonians for both these models (d) Model 2 (e) Comparison with simple SI model (f) effective Hamiltonian for both these models.

The kinetics of Model 1 is explained in Figure 1(a). Virus, *V*, can infect susceptible cells, *T*, with rate constant *k*, 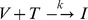. We neglect variations in the number of target cells and hence this reaction can be written as 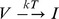. During its lifetime, an infected cell, *I*, continuously produces virus particles with rate 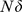, where *N* is the total number of infectious viral particles produced by a cell during its lifespan and 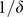 is the average lifespan of an infected cell. Virus particles are cleared and infected cells die, respectively, with rates 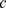 and 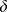.

#### Model 2 (Burst model)

Some viruses, rather than being produced continuously by an infected cell, leave the cell in a large burst that usually kills the cell as they exit. The kinetics of this model is explained in Figure 1(d). Once an infected cell dies, it releases *N>>1* virions in a single burst. The deterministic equations for the burst model (Model 2) and the continuous production model (Model 1) are identical. However, Pearson et al. [15] showed that the stochastic effects in these two models are different. Our coarse-graining approach will explain and quantify this observation naturally.

Initially we assume that the number of susceptible cells is much larger than the number of infected cells. In this case, we can neglect variations in the number of target cells and assume that 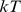 is a constant. This is the case that was previously considered by Pearson et al. [15]. We will explore it in the present work as well but this time we will use our new semiclassical coarse-graining approach.

At later stages of the infection, virus may infect many of the available susceptible cells and reach an equilibrium metastable state. This equilibrium depends on how new susceptible cells are produced or diffuse into the region of interest. Here we will consider a few examples by assuming that the total number of cells is kept constant, *N_t_*, so that *n_I_ = N_t_ – n_T_,.* where *n_I_* and *n_T_* are the numbers of infected and target cells, i.e., cells susceptible to infection, respectively.

The assumption of a constant total number of cells has been made in models of hepatitis B virus (HBV) and hepatitis C virus (HCV) infection of the liver [26], [27]. The liver is known to regenerate if it is damaged and a homeostatic process returns the liver to its original size. In HIV infection total CD4+ T cell numbers are not maintained constant over the disease course. However, since T cell depletion occurs on a time scale of years, with the T cell count on average falling from 1000/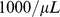 to 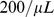 over about 10 years. Thus, over shorter periods, say days to months, the CD4+ T cell level is relatively constant in the chronic stage of disease. Thus, this approximation may be valid in such circumstances. We will explore the case in which the total cell population is held constant in the present work and use our semiclassical coarse-graining approach to calculate the probability to escape from this metastable state.

We will use the mean field assumption that in the considered region all uninfected cells are equally susceptible to infection by all virions. This assumption, however, does not restrict us in choosing a dependence of the viral infection rate 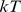 on the total number of cells *N_t_*, in the region of interest. There are two types of dependence, which emerge from different conditions. The first corresponds to a linear dependence of 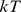 on the number of susceptible cells, i.e., 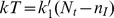, where 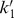 is a constant. This scaling is expected in a region where the law of mass-action should hold, i.e., a region that is well-mixed with fixed spatial dimensions and mobile susceptible cells and virions. Hence, we will discuss it in the context of HIV infection, in which virions and susceptible cells are assumed to be trapped in a finite region from which they are unlikely to escape. We note that the parameter 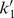 in 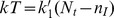 is an effective parameter that depends on the spatial dimensions of the considered region, since in a mass-action context 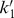 would be multiplied by a cell density, i.e. 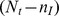 would be divided by the volume of the region and similarly 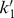 would implicitly need to be multiplied by this same volume. Since experimental data on infection kinetics within a region of known volume are not presently available, for demonstration of our method, we will use effective parameters that were previously used by Pearson et al. [15] in their studies of the early HIV infection.

Another scaling emerges when the region of interest contains a dense population of immobile cells, so that the spatial dimensions of the region increase with *N_t_*. In this case the probability of any given susceptible cell to be infected by a given virion decreases inversely proportional to *N_t_.* In such a case, we will assume 
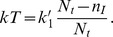
 We will consider this type of scaling during our discussion of HCV infection of cells in the liver. Here we note that in this case, the parameter 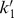 has a clear physical interpretation; it is the rate of collision of a virion with cells during the virion diffusion multiplied by the probability that upon collision with a susceptible cell that cell becomes infected. Because we assume 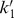 is constant, i.e. the virus collides with cells at a constant rate, the size of the region under consideration must be small. As we shall show, the value of 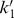 do not depend on the spatial dimensions of the considered region, and hence estimates of this parameter can be obtained from clinical data about average virus kinetics. Thus, our calculations in this second case can be performed completely using only known facts about average kinetics of HCV infection.

### Deterministic Scenario

Before considering stochastic effects, we note that the deterministic equations for the concentration of virus, *V,* and infected cells, *I,* in Model 1 can be written in the following form:
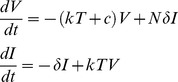
(1)


**Figure 2:**
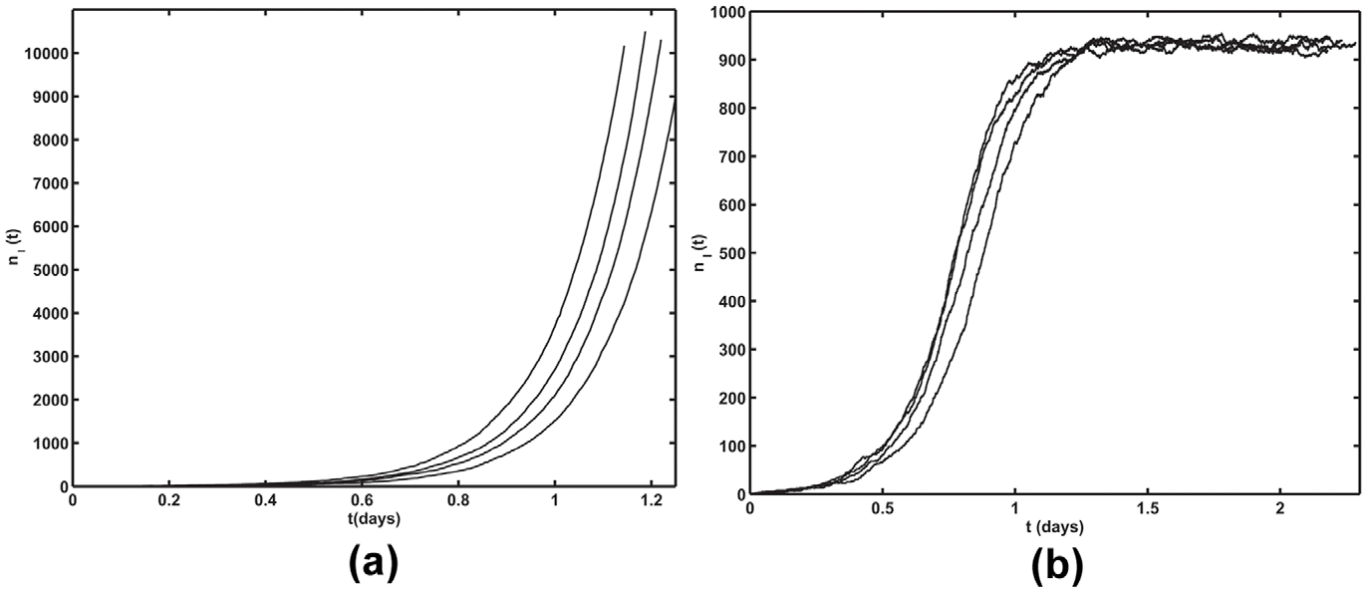
The number of infected cells *n_I_* versus time *t*, for Model 1 with multiple stochastic runs (a) *kT* =  constant, (b) 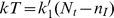. *n_I_* (0) = 0, *n_V_* (0) = 10, 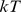 = 10 day^−1^, *N* = 30, 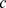 = 20 day^−1^, 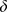 = 1 day^−1^, *N_t_* = 1000, and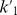 = 0.01 day^−1^.

Figure 2(a) shows the evolution of the number of infected cells at constant kinetic rates using Gillespie’s stochastic simulation method [28]. We use the following parameter values 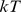 = 10 day^−1^, 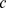 = 20 day^−1^, 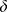 = 1 day^−1^, previously used by Pearson et al. [15] in their numerical simulations of HIV dynamics during the initial stage of infection, but set *N* = 30. The actual number of infectious virions released during chronic infection is not known. Chen et al. [29] estimate that in SIV infection about 50,000 SIV particles are released, but the fraction that are infectious has been estimated to be between 1 in 1,000 to 1 in 10,000, suggesting that *N* should lie between 5 and 50 [15]. However, recent estimates of the basic reproductive number, *R_0_*, for HIV suggest each infected cell infects about 10 others [30], and *N* would necessarily have to be 10 or larger, and thus we have set *N* = 30. In these simulations, the number of infected cells grows exponentially with time. Figure 2(b) shows that if the depletion of susceptible cells is taken into account (see Text S3), so that *kT* is no longer constant, 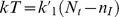, the number of infected cells reaches a steady state.

### Fluctuations

The dynamics of a stochastic system can be described by the birth- death master equation for the transition probabilities. For Model 1, the master equation for the probability of having 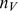 viruses and 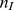 infected cells at time *t* is given by
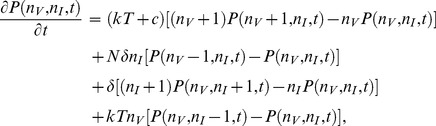
(2)and for Model 2 the master equation is given by



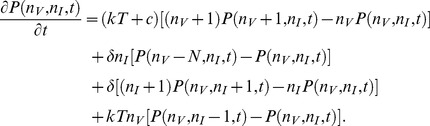
(3)


Solving these master equations would provide a complete description of the time evolution of the stochastic system, but in general it is impossible to obtain explicit solutions for the master equation.

### Semi-classical Treatment of the System’s Dynamics

For our models, it is impossible to get a closed form solution for master equations, and reasonable approximations are needed. Here we will employ an eikonal approximation to recast the problem in terms of an effective classical Hamiltonian system. This semi-classical method can be applied to simplify analysis of fluctuations, including rare event statistics, when the number of interacting objects is relatively large. This method has been used earlier to calculate the rare event statistics in reaction diffusion systems [16] and it has been applied to various epidemiological stochastic models (SI, SIS, SIR) as examples [18], [19].

Our goal is to find *the generating function*, 
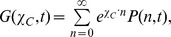
 where *n* is the vector of populations. Components of this vector are numbers of agents (i.e. virus particles, infected cells) of each type participating in a process. For example, in our models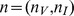, where 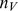 is the number of virions and 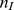 is the number of infected cells. Note that with every vector of populations, we associate a vector of conjugated variables, 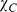. In our models, 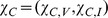. Knowledge of the generating function provides information about all measurable characteristics, including the extinction probability.

The master equation can be transformed into a Schrödinger-like equation for the evolution of *G* with effective quantum Hamiltonian, 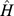 [16].
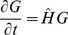
(4)


Here we do not provide the derivation of the semi-classical approach and refer the reader to Text S1 as well as original reviews and publications [16], [18], [31]. We only summarize the procedure for obtaining the generating function by this technique:

1. The method starts with using the eikonal ansatz 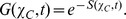

2. By considering the trajectories that dominate the dynamics, it was found that 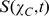 is given by

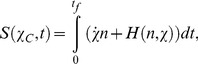
(5)where 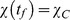 and the function *H,* which we will call the *Hamiltonian,* is obtained as follows:

Every elementary reaction contributes a specific term to *H*. For example, if a reaction corresponds just to a creation of particles with time according to a Poisson distribution, 
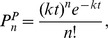
 then a corresponding term in the Hamiltonian is 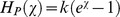. There are other rules that we will need in this article [20], [21], [31]:

2a) Let us consider a reaction describing a Poisson process of conversion of particles of the type *A* into particles of the type *B* given by.
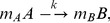
(6)


with some rate, *k*, which may depend on the number of all types of particles in the reaction diffusion system. The corresponding term in the Hamiltonian is given by.
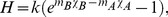
(7)


where 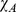 and 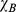 are conjugate variables to *A* and *B*. A detailed derivation of Eq. (7) via a stochastic path integral approach is provided in Text S1.

2b) When *m_B_* is not a constant, but some random variable with a distribution *P*(*m_B_*), then 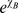 in Eq. (7) is replaced by 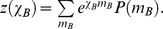


The latter rule follows from the fact that the Hamiltonian can be derived from the form of the generating function, 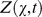, of the given reaction at *constant* parameters [20], [21], i.e. 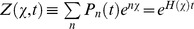. That is, if we find 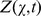 with an assumption that concentrations of particles are constant, we can identify the Hamiltonian of the reaction. A Poisson process that creates bursts of particles, with rate *k* and with each burst described by the function 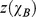 defined in the rule 2b), has the generating function 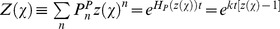. More rules about constructing terms in the Hamiltonian that correspond to various types of reactions can be found in earlier publications [20], [21], [31].

3.Vectors, *n*(*t*) and 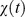 in Eq. (5), are obtained by solving the Hamiltonian equations.
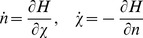
(8)


with boundary conditions, 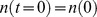 and 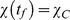. We can then substitute the solutions of Eq. (8) into Eq. (5) to obtain the generating function.

Following the above rules, (Eq. (6–7)) and examination of each term in Model 1, the Hamiltonian is given by.
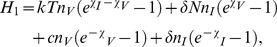
(9)


where there are four terms corresponding to each elementary reaction in Figure 1(a).

For Model 2 (burst model of virus production), the Hamiltonian is the sum of three terms.
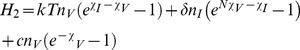
(10)


The latter Hamiltonian, *H*_2_, was derived assuming that number of viruses produced per infected cell is a constant. Following the rules described in 2b), if *N* is the mean of a Poisson distributed number of produced viruses, the Hamiltonian for Model 2 should rather be written as.
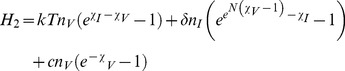
(11)


Note that the second term in *H_2_* follows from the rule 2b), where 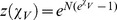 is the generating function of Poisson distributed virus particles with mean *N*. Hamiltonian equations of motion in both cases are given by.
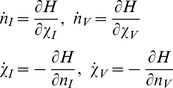
(12)


## Results

### Coarse-Graining

Equations. (9), (11) and (12) are a substantial simplification compared to the master equation because they are a set of a few ordinary differential equations in comparison to master equation, which is an infinite set of coupled differential equations for the probabilities of all possible events. In comparison to similar equations for SIS models, Eqs. (9) and (11) are still relatively complex and cannot be solved explicitly in a closed form. To resolve this problem, we note that in many applications, virus kinetics and kinetics of living cells are characterized by different time scales, namely, a single virus particle, when it is not inside an infected cell, is cleared from the body on a much faster timescale than the lifetime of an infected cell [1], [2], [4]. To be able to propagate, virus should multiply in large numbers (*N>1*) and infect cells as quickly as possible. Thus virus dynamics can often be considered fast in comparison with infected cell dynamics.

The approach of using such a time-scale separation in the semiclassical equations was developed previously [20], [21]. The idea is that fast degrees of freedom can be considered equilibrated at current values of slow variables, so that time-derivatives in Eq. (12) of the fast variables can be set to zero. The approach is commonly used in viral dynamic modeling and in that field is called a quasi-steady state assumption [32]. In particular, considering virus clearance to be fast, we can eliminate the virus-related variables by setting.
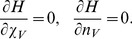
(13)


This reduces some of the differential equations to algebraic equations that can be solved explicitly, i.e., from Eq. (13) we obtain 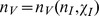 and 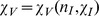. For example, for Model 1, by the elimination of the fast dynamics of the virus,
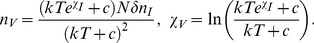
(14)


We then substitute this result back into the expression for the Hamiltonian and obtain the *effective Hamiltonian* that describes the lower-dimensional evolution of slow variables only. For Model 1, the effective Hamiltonian reduces to.
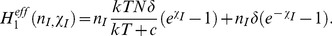
(15)


According to Eq. (15) and rules 2a-2b, Model 1 reduces to a previously extensively studied SIS model as shown in Figure 1(b) in which an infected cell either produces one more infected cell or dies, and both processes are exponentially distributed with time, as we illustrate in Figure 2(a). For this SIS model shown in Figure 1(b), one can write the master equation which can be solved exactly for the mean and variance using the generating function technique. These results can be used further to make estimates of the key rate parameters from experimental data that describe the primary phase of a viral infection even for a single sample as shown in Text S2 and Figure S1.

The effective Hamiltonian for Model 2 with a Poisson distribution of burst sizes, Eq. (11), after elimination of the fast viral degrees of freedom is given by.
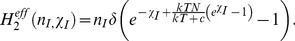
(16)


The form of *H_2_^eff^* in Eq. (16) corresponds to the decay of infected cells with rate 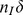, which when this happens, leads to an elimination of one infected cell and creation of a burst of new infected cells. Actually, Eq. (16) corresponds to the process, in which there is a burst release of free viruses which then either quickly decay or infect new cells. The number of newly infected cells per burst has Poisson statistics. Hence, a branching stochastic process Model 2 is reduced to an effective one as described in Figure 1(e). If a fixed burst size is assumed then starting from Eq. (10) after eliminating fast variables one obtains.
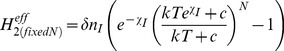
(17)


The two Hamiltonians, *H_1_^eff^* and *H_2_^eff^* describe evolutions of the number of infected cells in Models 1 and 2. The different forms of *H_1_^eff^* and *H_2_^eff^* mean that the behavior of fluctuations in the two models is very different. Nevertheless, the Hamiltonians, *H_1_^eff^* and *H_2_^eff^*, lead to essentially the same type of behavior for the mean number of infected cells. It is well known that the equation for an average quantity is given by [16], [17], [18], [31]:
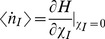
(18)


Then, for constant parameters, Eqs. (15), (16) and (17) all predict that.
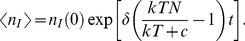
(19)


In this section, we showed that using the coarse graining approach [20], [21] it is possible to reduce the complexity of models of viral infections. The resulting Hamiltonian dynamics can be analyzed with the same approach as in previously studied SI models (see Text S3, Figure S2, for a model that includes susceptible cells, i.e. target cells, in addition to virions and infected cells).

Effective Hamiltonians can be used further to study infection kinetics on a coarse-grained level. For illustration, we will consider both models as examples and use the coarse graining technique to calculate disease extinction time.

### Spontaneous Infection Clearance

The extinction of a disease occurs along a trajectory in the phase space of the classical Hamiltonian known as the optimal path trajectory [33]. The probability of disease extinction decreases exponentially with increasing population size. The mean extinction time of the disease along the Hamiltonian trajectories that start from the metastable state and end at the extinction state where the number of infected cells reaches zero has the form.
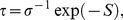
(20)


where *S* is known as *action* in classical physics [31]. A precise estimation of the prefactor σ can be a hard task because it is influenced by the non-semiclassical and geometric phase corrections to our approximations. Its precise value is, however, not important because Eq. (20) is dominated by the exponent. Using arguments in Reference [13] one can estimate that 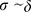.

As was discussed earlier, disease extinction is a rare event. Due to an unusually strong fluctuation the system can jump from its equilibrium metastable state to the extinction state along an optimal path that minimizes the action *S*. The optimal Hamiltonian trajectory that describes *S* starts from the metastable state and ends at the extinction state. Since the probability of extinction is found by minimizing the action, we compute such trajectories satisfying the zero energy condition, i.e., *H* = 0, as discussed in Reference [13]. This strongly simplifies further calculations because virus degrees of freedom are already eliminated, and one does not have to solve Hamiltonian equations explicitly. Instead, from *H* = 0, one can find how *n_I_* depends on 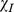 along this trajectory and then the action has the form.
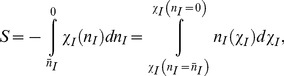
(21)


where 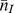
is the number of infected cells in the steady state.

At the metastable state, the average number of infected cells is given by.
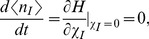
(22)


such that 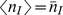


Consider the problem of spontaneous infection clearance from the metastable state in Model 1 and Model 2.

We first assume a finite well-mixed population of virions and cells such that 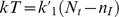, where 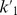 is a constant, and *N_t_* is the total number of cells.

### Model 1

Following the above methodology, the number of infected cells in the steady state for Model 1 is.
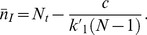
(23)


Using the relation 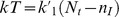 and equating the effective Hamiltonian for Model 1 to zero we get the following algebraic equation for the number of infected cells.
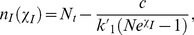
(24)


where 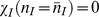 and 
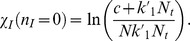
 Substituting Eq. (24) into Eq. (21) one can calculate the action *S* and from it the mean extinction time according to Eq. (20).
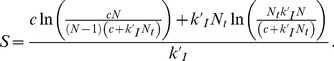
(25)


### Model 2

Similarly, following Eq. (22).
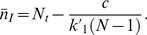
(26)


Again equating the Hamiltonian for Model 2 to zero yields.
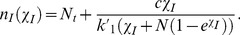
(27)


Substituting this into Eq. (21), *S* can be computed numerically.

**Figure 3:**
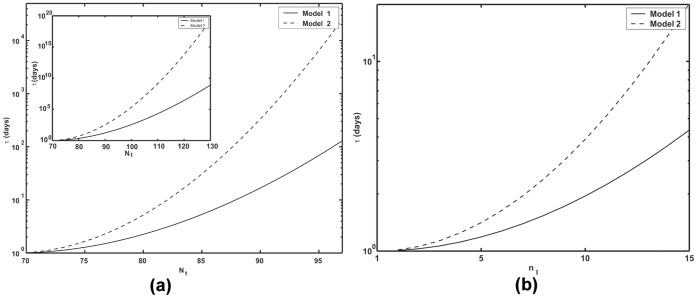
Numerically computed mean extinction time 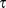, (a) as a function of *N_t_*, inset shows the extinction time at higher values of *N_t_* (b) as a function of *n_I_*, solid line: Model 1 and dashed line: Model 2, 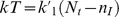, *N* = 30, 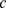 = 20 day^−1^, 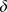 = 1 day^−1^ and 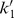 = 0.01 cell^−1^ day^−1^.

Figure 3(a) shows the numerically computed mean extinction time 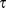 as a function of *N_t_* for both of these models with parameter values applicable to HIV dynamics. While the two models predict quantitatively different exponents, qualitatively they lead to similar conclusions at realistic parameter values. For a total population of around 70 to 80 cells in an isolated region, one is likely to see spontaneous infection clearance within several days. As a practical matter spontaneous extinction can take place only up to a critical cell number. As *N_t_* increases further, the extinction times become so large that they become unreachable, suggesting that the infection cannot be cleared spontaneously at those conditions. From Eq. (24) or (27) one can compute that for the parameters used in Fig. 3, when *N_t_* = 80, the number of infected cells is about 11. Thus spontaneous clearance in a few days would only be expected to occur in small foci of infected cells of order 10 or less as shown in Figure 3(b). Further, as one can see from the figure spontaneous clearance is more rapid when virus production is continuous (model 1) than when it occurs in bursts (model 2).

Let us now switch to the case for which 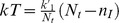. One can calculate the mean extinction time for both the models using the same methodology as above:

### Model 1



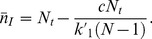
(28)

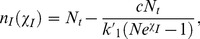
(29)


where
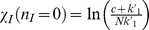
and 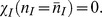


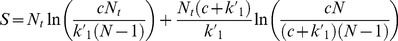
(30)


### Model 2

Using Eq. (22) and equating the Hamiltonian in Eq. (16) to zero, we get.
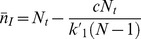
(31)

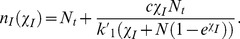
(32)


The action *S* can then be calculated numerically using the above set of equations.

**Figure 4:**
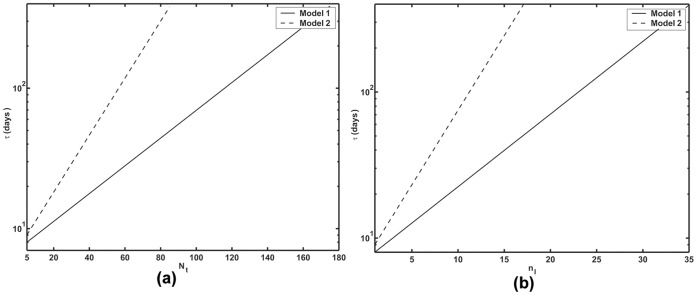
Numerically computed mean extinction time 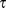, (a) as a function of *N_t_* (b) as a function of *n_I_*, Solid line: Model 1 (solid line) and dashed line: Model 2, 
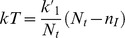
, *N* = 336 virions, 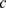 = 6.2 day^−1^, 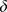 = 0.14 day^−1^, and 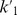 = 0.0231 day^−1^.

From the action, *S,* one can numerically calculate the mean extinction time, 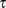, as a function of *N_t_*, this time, in the context of HCV dynamics where 
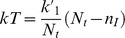
. Unlike HIV and HBV, HCV is a chronic infection that can be cured in patients by antiviral therapy [2], [34]. Spontaneous clearance of HCV infection has also been reported, both in the absence and presence of HCV-specific cellular immune responses [11], [26], [35], and our theory can make predictions about the size of regions in the liver that can be cleared spontaneously. It has been estimated that in the absence of treatment about 5%–20% of hepatocytes (liver cells) are infected during chronic HCV infection [36], [37]. Taking the geometric mean of these estimates, we shall assume 10% of hepatocytes in any region of the liver are infected. Further, as done by Rong et al. [34], we assume that due to different states of differentiation or due to some cells being in an interferon-induced “antiviral state” only about 50% of hepatocytes are targets of HCV infection at any time (i.e., *N_t_* is about 50% of hepatocytes in any region being considered and hence 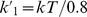). Then using previously estimated parameter values characterizing HCV infection, *c* = 6.2 day^−1^, 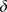 = 0.14 day^−1^, *N* = 336, *kT* =  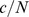 [34], we find 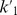 = 0.023 days^−1^. Figure 4(a) shows the numerically calculated mean extinction time, 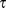, as a function of *N_t_* for models 1 and 2. We predict for models 1 and 2, for regions of the liver that contain around 170 and 85 susceptible hepatocytes (Figure 4(a)) or around 380 and 170 total hepatocytes, respectively, infection can be cleared spontaneously within a year. If *N_t_* >170 for model 1 and *N_t_* >85 for model 2, spontaneous infection clearance becomes practically impossible. With 10% of the total hepatocytes being infected, for model 1 and 2, within a year, one is likely to see spontaneous infection clearance in a small region with around 35 or 17 infected cells respectively (Figure 4(b)). It is also seen from Figure 4(b) that 10 or fewer infected cells are removed from the human liver spontaneously in about 20 days, while for HIV infection 10 infected cells can be spontaneously cleared in about 4 days (Figure 3(b)). Also, as in the case of HIV, clearance is more rapid in model 1 than in model 2. Also, for HCV continuous production model (model 1) is probably more biologically realistic than burst production as there is little evidence to suggest that the virus kills infected cells in vivo.

Ordinary differential equation models of HCV infection and treatment have shown that there is a critical drug effectiveness, 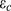, such that if the drug effectiveness, 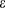, is below 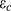
_,_ the HCV viral load goes to a new on therapy steady state in which the HCV level is lower than in the pre-treatment steady state [38]. The drug effectiveness can be below its critical value when therapy is suboptimal as appears to be the case in the approximately 50% of people who do not clear HCV with interferon-based therapy. In addition, 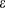 may be less than 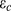 in regions of the liver where drugs do not reach properly or in the presence of drug resistant HCV variants. Our calculations suggest that viral clearance may still occur when 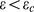
due to spontaneous fluctuations. The closer 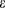 is to 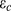
_,_ the lower the on therapy viral load and the more likely spontaneous fluctuations can clear the infection. We note, however, that the presence of such regions in HCV infected liver in which clearance is spontaneous remains a hypothesis.

We also make an observation that the exponent, *S*, in Eq. (20) for the mean time to clearance is independent of the parameter 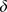, which represents the infected cell death rate. One can show that the latter rate only enters the prefactor of the exponent, 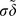. Thus the kinetics of infection clearance is not very sensitive to this rate. Physically this means that infection clearance is not exponentially sensitive to the average lifetime of an infected cell. In contrast, we found that the infection lifetime is exponentially sensitive to the parameters 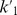, *N* and *c*, all of which describe virus kinetics.

## Discussion

We have developed a theory based on a mixed computational/theoretical approach to analyze stochastic events in the course of a viral infection. This method has been applied earlier to stochastic biochemical networks, such as enzymatic reactions, characterized by slow non- Poissonian fluctuations [20].

We explored two previously introduced models of viral infection that involve either continuous or burst production of virus from infected cells and used the coarse-grained semi-classical treatment to capture various aspects of the corresponding stochastic dynamics. During any viral infection, from HIV to the common cold, it is important to know whether the infection goes extinct or becomes persistent. We predicted the mean time for infection clearance in both our models and showed that this extinction time is sensitive to virus related parameters such as the virion clearance rate and the virion infectivity. On the other hand, the extinction time is not exponentially sensitive to the lifetime of an infected cell. This is in contrast to deterministic evolution models, in which all kinetic rate parameters are usually equally important and the time to eliminate an infection by therapy is more sensitive to the lifetime of an infected cell than other parameters [39]. Thus, if drug therapy fails to clear an infection and leaves foci of infected cells in tissues, as has been suggested for both HIV and HCV [13], [40], then intensifying therapy by adding a drug that reduces infection would seem to be a better strategy that adding an immune modulator that could increase the rate of infected cell death.

Our theoretical approach provides a method to estimate of the number of cells in a finite population that can be cleared spontaneously leading to disease extinction. For parameters relevant to HIV infection, as used here, we showed that spontaneous clearance is only likely to occur in small cell populations, i.e., a population of less than 100 cells (Figure 3(a)). Thus, once infection is established throughout the body, spontaneous extinction is predicted not to occur, consistent with the observation that essentially no one infected with HIV has spontaneously cleared the infection. Nonetheless, the theory predicts that if small isolated clusters of infected T cells exist, infection could spontaneously die out in individual clusters. Note the simple theory presented here, ignores the complications of latently infected cells [41] and long-lived infected cells [42], the existence of which reduce the probability of extinction. However, our theoretical method can be straightforwardly generalized and applied to more complex interacting stochastic processes, such as those involving these additional cell populations, as well as situations in which neither the number of target cells nor the total number of cells are assumed to be constant.

In the case of HCV infection, the assumption of a constant total number of cells, susceptible plus infected, has been made previously and seems reasonable as this virus, unlike HIV, usually does not kill the cells it infects. Further, unlike HIV, spontaneous clearance of infection has been frequently observed [6] and in some cases even in the absence of a strong immune response [11]. For parameters relevant to HCV infection, we showed that spontaneous clearance can occur in infected cell populations of the order of 40 infected cells or less in a region of the human liver, based on predictions of the continuous production model (Figure 4(b)). It is unclear if current drug therapy completely clears HCV infection or simply reduces the amount of virus and number of infected cells to a low level, with the ultimate clearance then being immune mediated or occurring spontaneously due to stochastic fluctuations, as in our model [40]. However, more elaborate models that incorporate immune defenses would be valuable to pursue in the context of the stochastic modeling approach presented here.

## Supporting Information

Figure S1 The logarithm of *n_I_*(*t*)/*n_I_*(0) as a function of time *t*. The numerically simulated data points for the number of infected cells *n_I_* at equal time intervals is given by the black points while the solid curve is a linear fit to the simulation data points.
(TIF)

Figure S2 Schematic representation of (a) viral infection model (b) simple SI model (c) comparison between their effective Hamiltonians.
(TIF)

Text S1 (DOC)

Text S2 (DOC)

Text S3 (DOC)
