# Supplementary figures and images for: Spontaneous Clearance of Viral Infections by Mesoscopic Fluctuations
Source: PLoS One. 2012 Jun 5;7(6):e38549. doi: 10.1371/journal.pone.0038549 (PMC3367925; doi:10.1371/journal.pone.0038549)

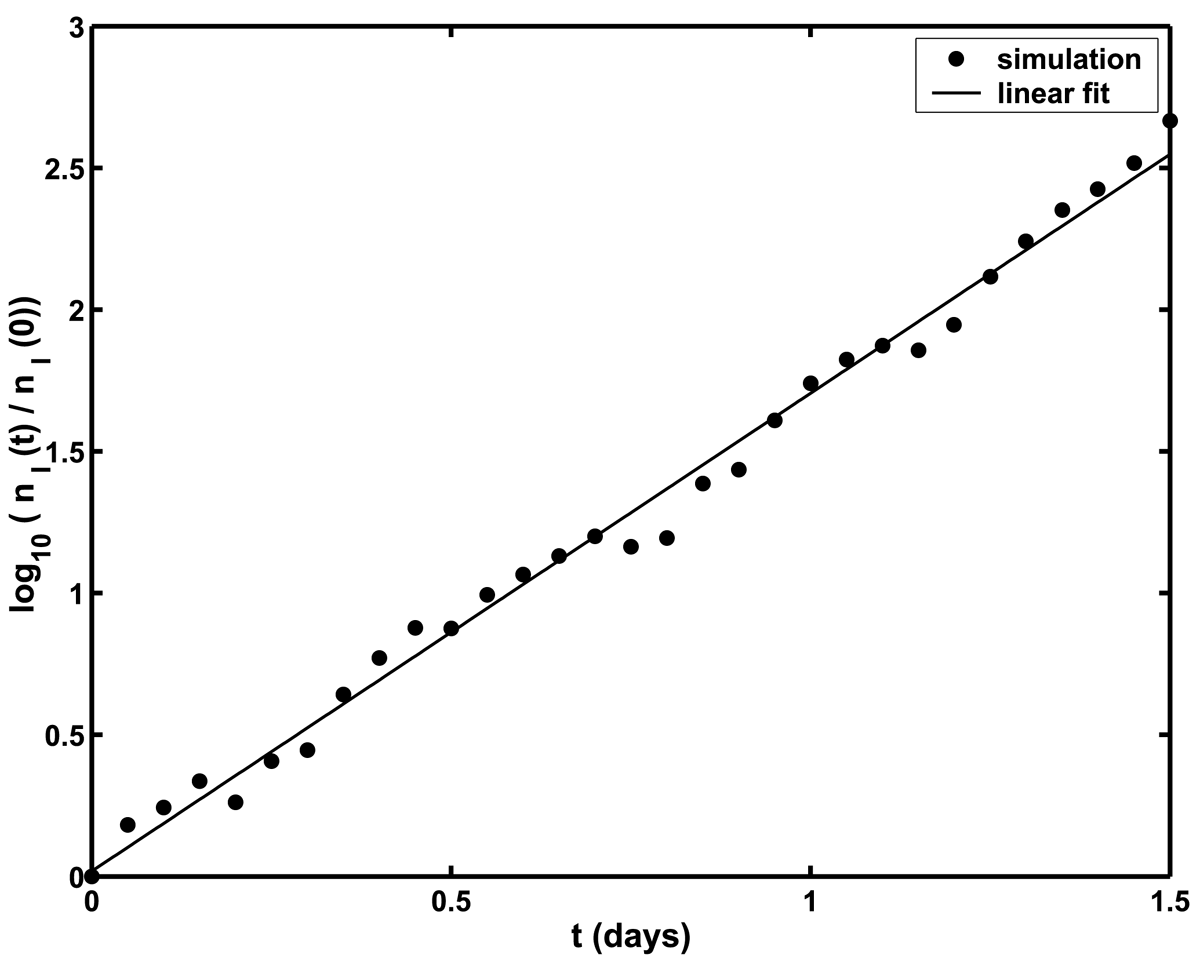

Supplement: Figure S1 — The logarithm of nI(t)/nI(0) as a function of time t. The numerically simulated data points for the number of infected cells nI at equal time intervals is given by the black points while the solid curve is a linear fit to the simulation data points. (TIF) [file pone.0038549.s001.tif]

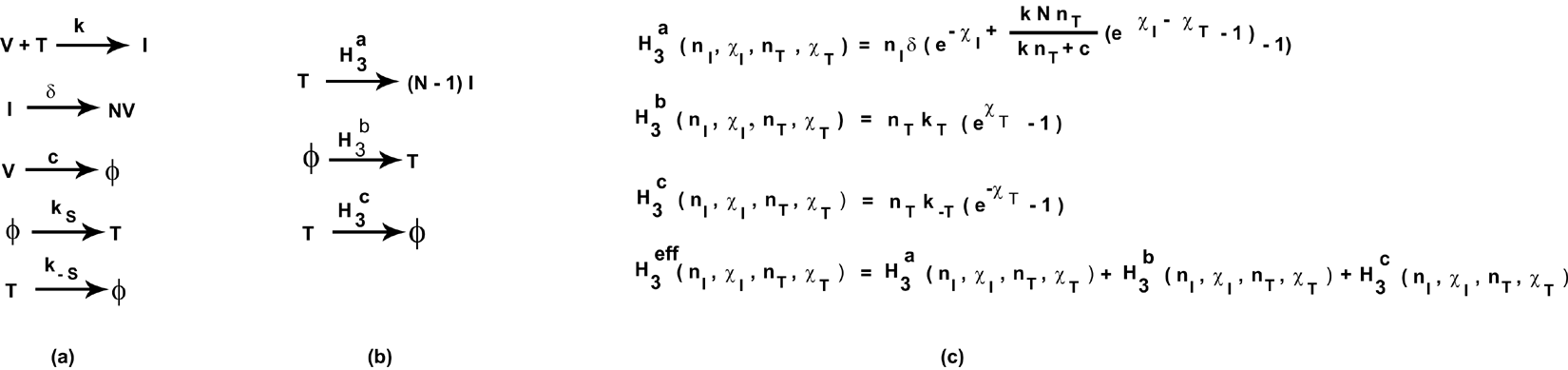

Supplement: Figure S2 — Schematic representation of (a) viral infection model (b) simple SI model (c) comparison between their effective Hamiltonians. (TIF) [file pone.0038549.s002.tif]
