# Supplementary material for: Spontaneous Clearance of Viral Infections by Mesoscopic Fluctuations
Source: PLoS One. 2012 Jun 5;7(6):e38549. doi: 10.1371/journal.pone.0038549 (PMC3367925; doi:10.1371/journal.pone.0038549)
Supplement: Text S1 — (DOC) [file pone.0038549.s003.doc]

**Supporting Information File (Text S1)**

Srabanti Chaudhury, Alan S. Perelson and Nikolai A. Sinitsyn

**STOCHASTIC PATH INTEGRAL (SPI) TECHNIQUE**

The stochastic path integral is used to simplify studies of the distribution function, including rare event statistics , in the case when number of interacting objects (e.g. infected cells) is much larger than one. Thus, a network of *M* reactions with *N* chemical species can generally be described by only 2*MN* ordinary differential equations using the SPI-technique . Thus, although the formal justification of the technique may look complicated the method for applications can be formulated as a set of rules that lead to a relatively simple set of deterministic ordinary differential equations. In this supplementary material we illustrate in detail the derivation of the rules for the stochastic path integral in the case of a reversible Poisson reaction. Further discussion of the rules can be found in the cited literature. Consider a simple process when one chemical *S* (substrate) is converted reversibly into another chemical *P* (product) with rates *k_sp_* and *k_ps_* respectively.

We introduce the moment generating function (MGF) for the total increase in the number of

product molecules *Q* during time *T*:

$$Z(\chi_{C})=e^{S\left(\chi_{C}\right)}=\sum_{Q=-\infty}^{\infty} P_{Q}e^{iQ\chi_{C}}$$

Note that *Q* can be negative, which corresponds to more reaction events in the reverse direction

than in the forward direction. We will also assume that the reaction rates depend on the number of particles of any type, i.e., *k_sp_* = *k_sp_*(*N_s_,N_p_*) and *k_ps_* = *k_ps_*(*N_s_*,*N_p_*), which make reactions non-Poisson. However, in the case of large particle numbers (*N_s_, N_p_ >>* 1) the relative change of concentrations after several reaction events can be considered negligible. This means that it is possible to pick the time scale $\delta t$ such that slow variables *N_s_* and *N_p_* can be considered constant on this time scale, and reactions can be considered Poisson distributed and independent of each other.

Let's pick such a time interval of size $\delta t$ around time point *t_k_* and introduce random variables$\delta Q_{sp}\left(t_{k}\right)$ and$\delta Q_{ps}\left(t_{k}\right)$, that are the numbers of substrate molecules converted to product and product molecules converted to substrate during this time interval. Formally, their probabilities can be written as an inverse Fourier transformation

$$P\left(\delta Q_{sp/ps}\left(t_{k}\right)\right)=\frac{1}{2\pi}\int d\chi_{sp/ps}\left(t_{k}\right)e^{-i\chi_{sp/ps}\left(t_{k}\right)\delta Q_{sp/ps}\left(t_{k}\right)+H_{sp/ps}\left(\chi_{sp/ps}\left(t_{k}\right),N_{s}\left(t_{k}\right),N_{p}\left(t_{k}\right)\right)\delta t}$$

where

$$H\left(\chi_{sp},N_{s},N_{p}\right)=k_{sp}\left(N_{s},N_{p}\right)\left(e^{i\chi_{sp}-1}\right), H\left(\chi_{ps},N_{s},N_{p}\right)=k_{ps}\left(N_{s},N_{p}\right)\left(e^{i\chi_{ps}-1}\right)$$

The MGF of number of product molecules created during time interval (0, *T*) is given by the path integral over all possible values of $\delta Q_{i}$.

$$\begin{matrix} \left\langle e^{i\chi_{C}Q}\right\rangle=\prod_{k} \int DN_{s}\left(t_{k}\right)DN_{p}\left(t_{k}\right)\int D\delta Q_{sp}\left(t_{k}\right)D\delta Q_{ps}\left(t_{k}\right)P\left[\delta Q_{sp}\left(t_{k}\right)\right]P\left[\delta Q_{ps}\left(t_{k}\right)\right]e^{i\chi_{C}\sum_{t_{k}} \left\{\delta Q_{sp}\left(t_{k}\right)-\delta Q_{ps}\left(t_{k}\right)\right\}} \\ \delta\left(N_{s}\left(t_{k+1}\right)-N_{s}\left(t_{k}\right)-\delta Q_{ps}\left(t_{k}\right)+\delta Q_{sp}\left(t_{k}\right)\right)\delta\left(N_{p}\left(t_{k+1}\right)-N_{p}\left(t_{k}\right)-\delta Q_{sp}\left(t_{k}\right)+\delta Q_{ps}\left(t_{k}\right)\right) \end{matrix}$$

Here we used the fact that $Q=\sum_{k} \left(\delta Q_{sp}\left(t_{k}\right)-\delta Q_{ps}\left(t_{k}\right)\right)$. The$\delta$-function in the path integral expresses the conservation law for the slowly changing number of substrate/product molecules.

In the next step we rewrite delta function as the inverse Fourier transform of the oscillating exponent

$$\begin{matrix} \delta\left(N_{s/p}\left(t_{k+1}\right)-N_{s/p}\left(t_{k}\right)-\delta Q_{ps/sp}\left(t_{k}\right)+\delta Q_{sp/ps}\left(t_{k}\right)\right) \\ =\frac{1}{2\pi}\int_{-\pi}^{+\pi} d\chi_{s/p}\left(t_{k}\right)\exp\left\{i\chi_{s/p}\left(t_{k}\right)\left(N_{s/p}\left(t_{k+1}\right)-N_{s/p}\left(t_{k}\right)-\delta Q_{ps/sp}\left(t_{k}\right)+\delta Q_{sp/ps}\left(t_{k}\right)\right)\right\} \end{matrix}$$

and substitute it together with (3) into (5). After this, integrations over $\delta Q_{sp/ps}\left(t_{k}\right)$ produce new

$\delta$-functions on $\chi_{sp/ps}$ that are easily removed by integration over $\chi_{i}\left(t_{k}\right)$ thus leaving us only with a path integral over slow $N_{s/p}\left(t\right)$ and $\chi_{s/p}\left(t\right)$variables.

$$\left\langle e^{i\chi_{C}Q}\right\rangle=\int DN_{s}DN_{p}\int D\chi_{s}D\chi_{p}e^{S\left(\chi_{C}\right)}$$

where

and $H_{sp/ps}$ are given in (4).

The large *N*-assumption allows us to make a standard transition from the path integral representation to the exponent of the classical action

$$\left\langle e^{i\chi_{C}Q}\right\rangle\approx e^{S\left(\chi_{C}\right)},$$

where now $\chi_{i}$ and *N_i_* are functions, satisfying the canonical equations of motion

These equations should be supplemented by a set of conditions on initial and final values of the variables *N_i_* and $\chi_{i}$. For example for initial conditions with the known number of particles *N_i0_* at the initial moment the boundary conditions are

$N_{i}\left(0\right)=N_{i0}, \chi_{i}\left(T\right)=0$.

The reason for introducing the imaginary unit in definition in Eq. (9) was to stress that the generating function in Eq. (9) becomes periodic function of counting field because *Q* is an integer. In many applications, this information is not important and one can get rid of imaginary unit by making a transformation $\chi_{i}\to i\chi_{i}$. Then Eq. (11) transfers to Eq. (8) in the main text. Moreover, in calculations of extinction time, the information encoded in parameter $\chi_{C}$is irrelevant, and one can set this constant parameter to zero.

The power of the semiclassical approximation Eqs. (9-11) is in the reduction of the partial differential equation for the generating function to the finite set of ordinary differential equations for the canonical variables *N_i_* and $\chi_{i}$. Note, however, that this approximation is still very rich in comparison with the standard mean field approximation that relies on the equation for average numbers of particles. In the semiclassical approach the number of independent variables is twice as large as in the mean field. Equations (9-11) contain information about the whole generating function $S\left(\chi_{C}\right)$.

The path integral representation of the chemical fluxes can be easily generalized to a network with an arbitrary number of chemicals and reactions. For a large number of reacting molecules there is a time scale $\delta t$ such that all fast reactions that connect different slow variables can be considered statistically independent; therefore in the path integral for the evolution of slow variables, every new reaction simply adds a separate contribution to the effective Hamiltonian and to the geometric part of the action.

Lets enumerate slow chemical species by the letters *i* or *j* and $H_{ij}\left(\left\{N\right\},\left\{\chi\right\},\left\{\chi_{C}\right\}\right)$ is the corresponding cumulant generating function for the reaction labeled by [*ij*] and where $\left\{N\right\}$, $\left\{\chi\right\}$ and $\left\{\chi_{C}\right\}$represent the full set of variables and counting fields in the network. Then the total effective action in the path integral representing the dynamics of the whole network is given by the expression

,

i.e., every chemical reaction adds a term in the effective "Hamiltonian" and every chemical species is described by a pair of canonically conjugated variables *N_i_* and $\chi_{i}$.

**References:**

1. Pilgram S, Jordan AN, Sukhorukov EV, Buttiker M (2003) Stochastic path integral formulation of full counting statistics. Phys Rev Lett 90: 206801.

2. Elgart V, Kamenev A (2004) Rare event statistics in reaction-diffusion systems. Phys Rev E 70: 041106.

3. Jordan AN, Sukhorukov EV, Pilgram S (2004) Fluctuation statistics in networks: A stochastic path integral approach. J Math Phys 45: 4386-4417.
