# Supplementary material for: Spontaneous Clearance of Viral Infections by Mesoscopic Fluctuations
Source: PLoS One. 2012 Jun 5;7(6):e38549. doi: 10.1371/journal.pone.0038549 (PMC3367925; doi:10.1371/journal.pone.0038549)
Supplement: Text S2 — (DOC) [file pone.0038549.s004.doc]

**Supporting Information File (Text S2)**

Srabanti Chaudhury, Alan S. Perelson and Nikolai A. Sinitsyn

Consider the model in Figure 1(b), i.e., the continuous viral production model with virus slaved to the infected cell level, with constant $a\equiv\frac{kTN\delta}{kT+c}$and $b\equiv\delta$. We assume *a* > *b* such that the population of infected cells is expected to grow. The master equation for the probability $P\left(n_{I},t\right)$ to have $n_{I}$ infected cells takes the simple form

$$\frac{\partial P\left(n_{I},t\right)}{\partial t}=b\left(n_{I}+1\right)P\left(n_{I}+1,t\right)-bn_{I}P\left(n_{I},t\right)+a\left(n_{I}-1\right)P\left(n_{I}-1,t\right)-aP\left(n_{I},t\right)$$

To solve this equation we introduce a probability generating function

$G\left(z,t\right)=\sum_{n_{I}=0}^{\infty} z^{n_{I}}P\left(n_{I},t\right)$,

where $z=e^{\chi_{I}}$. The fact that the probability is conserved requires the following normalization condition

$$G\left(1,t\right)=1$$

The master equation can be written in terms of the generating function as

$$\frac{\partial G\left(z,t\right)}{\partial t}=\left[az\left(z-1\right)+b\left(1-z\right)\right]\frac{\partial G\left(z,t\right)}{\partial z}.$$

This equation can be solved with the initial condition be $G\left(z,0\right)=z^{n_{I}\left(0\right)} ,$ where $n_{I}\left(0\right)$ is the initial number of infected cells, yielding

$$G\left(z,t\right)=\left(1+\frac{\left(a-b\right)\left(1-z\right)}{a\left(z-1\right)-e^{-\left(a-b\right)t}\left(az-b\right)}\right)^{n_{I}\left(0\right)} .$$

The moments of the probability distribution function (PDF) *P*(*n_I_*,*t*) can be obtained from the derivatives of the generating function at *z* = 1, e.g., the mean of the distribution is

$$\left\langle n_{I}\left(t\right)\right\rangle=\partial_{z}G\left(z=1,t\right)=n_{I}\left(0\right)\exp\left[\left(a-b\right)t\right],$$

MERGEFORMAT

the variance is

$$\left\langle n_{I}^{2}\left(t\right)\right\rangle-\left\langle n_{I}\left(t\right)\right\rangle^{2}=\frac{2an_{I}\left(0\right)e^{2\left(a-b\right)t}\left(1-e^{-\left(a-b\right)t}\right)}{a-b}+n_{I}\left(0\right)e^{\left(a-b\right)t}\left(1-e^{\left(a-b\right)t}\right),$$

and the extinction probability is given by

$$P_{ext}=G(0)=\left(1-\frac{(a-b)}{a-be^{-(a-b)t}}\right)^{n_{I}(0)}.$$

Observation of stochastic fluctuations during infection growth can be used to learn more about the virus kinetics. Imagine, that we perform measurements of the number of infected cells, for a single patient or tissue sample, at equal time intervals τ. Let’s assume that we know that Model 1 is correct but we do not know values of the coefficients, *a* and *b*. We performed Gillespie simulations to generate a stochastic trajectory for the number of infected cells *n_I_*(*t*) corresponding to this process. What can we learn about those coefficients by making such a measurement *of a single stochastic trajectory*?

First, the solution of the deterministic equations for this model predicts the population of infected cells,$n_{I}$, grows exponentially so that $n_{I}\left(t\right)=n_{I}\left(0\right)\exp\left[\left(a-b\right)t\right]$. The slope of the logarithm of *n_I_*(t)/*n_I_*(0), gives an estimate of the coefficient, *a-b*, that is $\delta\left(\frac{kTN}{kT+c}-1\right)$ in Model 1. This fixes one combination of parameters but we still cannot determine *a* and *b* separately. Let us now calculate the variance of the number of infected cells during a time interval, *τ*. Let $n_{I}\left(i\right)$ be the number of infected cells at each time interval. According to the exact solution in Eq. (1) we have

$$\left\langle\left(n_{I}\left(i+1\right)-n_{I}\left(i\right)\right)^{2}\right\rangle=n_{I}\left(i\right)\frac{2ae^{2\left(a-b\right)\tau}\left(1-e^{-\left(a-b\right)\tau}\right)}{a-b}+n_{I}\left(i\right)e^{\left(a-b\right)\tau}\left(1-e^{\left(a-b\right)\tau}\right)$$

We can then define a quantity$X_{i}=\left(n_{I}\left(i+1\right)-n_{I}\left(i\right)\right)^{2}/n_{I}\left(i\right)$, such that

$$\left\langle X\right\rangle=\sum_{i=1}^{N-1} X_{i}=\frac{2ae^{2\left(a-b\right)\tau}\left(1-e^{-\left(a-b\right)\tau}\right)}{a-b}+e^{\left(a-b\right)\tau}\left(1-e^{\left(a-b\right)\tau}\right).$$

where *N* is the number of points taken at equal time intervals τ. One can see that all *X_i_* have the same mean value, which we can estimate using measurements of <*X*> in a single trajectory. Using this relation one can get an estimate of the parameters *a* and *b* for Model 1 separately. We note that those two combinations also control the extinction time of infection, as we will show below. Figure S1 shows *n_I_*(*t*) for the continuous model calculated at equal time intervals (black dots). We started with 10 virions and 10 infected cells and generated 1000 realizations until we found one with around 1,000 cells are infected. We then extracted and plotted 31 simulation data points (*N*= 31) at equal time intervals of *τ =* 0.05. Due to stochastic fluctuations, all the data points (black dots) do not lie exactly on the linear fit to the simulation data points. Using Eq. (10), and the parameter values from Figure S1, we estimate *a*= 3.33/day and *b*= 1/day. Thus observation of stochastic fluctuations in infection growth allows one to estimate parameters of infection that cannot be obtained by using only comparison with deterministic models.

References:

1. Gardiner CW (1996) Handbook of Stochastic Methods: For Physics, Chemistry and the Natural Sciences. New York: Springer.

2. Gillespie DT (1977) Exact stochastic simulation of coupled chemical reactions. J Phys Chem 81: 2340–2361.

**Figure Legend**

S1. The logarithm of *n_I_*(*t*)/*n_I_*(0) as a function of time *t*. The numerically simulated data points for the number of infected cells *n_I_* at equal time intervals is given by the black points while the solid curve is a linear fit to the simulation data points.
