# Supplementary material for: Spontaneous Clearance of Viral Infections by Mesoscopic Fluctuations
Source: PLoS One. 2012 Jun 5;7(6):e38549. doi: 10.1371/journal.pone.0038549 (PMC3367925; doi:10.1371/journal.pone.0038549)
Supplement: Text S3 — (DOC) [file pone.0038549.s005.doc]

**Supporting Information File (Text S3)**

Srabanti Chaudhury, Alan S. Perelson and Nikolai A. Sinitsyn

**General viral SI model**

Here we point out that the coarse-graining approach can be easily applied to more general models. For example, consider Model 3 in Figure 3(a), which represents the situation that involves susceptible cells in addition to virions and infected cells, which all have their own kinetics. This set of reactions is a more general case of the Model 2. Its Hamiltonian is given by

where *n_T_* is the number of susceptible cells. Considering the case in which the burst size *N* has a Poisson distribution and eliminating the fast degrees of freedom of the virus, the effective Hamiltonian then is given by

$$H_{3}^{eff}\left(n_{I,}\chi_{I},n_{T},\chi_{T}\right)=n_{I}\delta\left(e^{-\chi_{I}+\frac{kn_{T}N}{kn_{T}+c}\left(e^{\chi_{I}-\chi_{T}}-1\right)}-1\right)+k_{-T}n_{T}\left(e^{-\chi_{T}}-1\right)+k_{T}\left(e^{\chi_{T}}-1\right).$$

This is similar to well-known SI model that divides the host into two dynamic subpopulations, the infected and the susceptible cells, described in Figure 3(b).

Figure legend

S2. Schematic representation of (a) viral infection model (b) simple SI model (c) comparison between their effective Hamiltonians.
